# Resistance Training Combined With Stretching Increases Tendon Stiffness and Is More Effective Than Stretching Alone in Children With Cerebral Palsy: A Randomized Controlled Trial

**DOI:** 10.3389/fped.2019.00333

**Published:** 2019-08-13

**Authors:** Barbara M. Kalkman, Gill Holmes, Lynn Bar-On, Constantinos N. Maganaris, Gabor J. Barton, Alfie Bass, David M. Wright, Roger Walton, Thomas D. O'Brien

**Affiliations:** ^1^INSIGNEO Institute for in silico Medicine, University of Sheffield, Sheffield, United Kingdom; ^2^Alder Hey Children's NHS Foundation Trust, Liverpool, United Kingdom; ^3^Department of Rehabilitation Medicine, Amsterdam Movement Sciences, Vrije Universiteit Amsterdam, Amsterdam, Netherlands; ^4^Department of Rehabilitation Sciences, Katholieke Universiteit Leuven, Leuven, Belgium; ^5^Research Institute for Sport and Exercise Science, Liverpool John Moores University, Liverpool, United Kingdom

**Keywords:** stretching, cerebral palsy, fascicle, ultrasound, tendon, stiffness

## Abstract

**Aim:** Stretching is often used to increase/maintain muscle length and improve joint range of motion (ROM) in children with cerebral palsy (CP). However, outcomes at the muscle (remodeling) and resulting function appear to be highly variable and often unsatisfactory. During passive joint rotation, the Achilles tendon lengthens more than the in-series medial gastrocnemius muscle in children with CP, which might explain the limited effectiveness of stretching interventions. We aimed to ascertain whether increasing tendon stiffness, by performing resistance training, improves the effectiveness of passive stretching, indicated by an increase in medial gastrocnemius fascicle length.

**Methods:** Sixteen children with CP (Age median [IQR]: 9.6 [8.6, 10.5]) completed the study. Children were randomly assigned to a combined intervention of stretching and strengthening of the calf muscles (*n* = 9) or a control (stretching-only) group (*n* = 7). Medial gastrocnemius fascicle length at a resting ankle angle, lengthening during passive joint rotations, and tendon stiffness were assessed by combining dynamometry and ultrasound imaging. The study was registered on clinicaltrials.gov (NCT02766491).

**Results:** Resting fascicle length and tendon stiffness increased more in the intervention group compared to the control group (median [95% CI] increase fascicle length: 2.2 [1.3, 4.3] mm; stiffness: 13.6 [9.9, 17.7] N/mm) Maximum dorsiflexion angle increased equally in both groups.

**Conclusion:** This study provides proof of principle that a combined resistance and stretching intervention can increase tendon stiffness and muscle fascicle length in children with CP. This demonstrates that remodeling of muscle structure is possible with non-invasive interventions in spastic CP.

## Introduction

Cerebral palsy (CP) is a non-progressive disorder caused by a brain lesion in early stages of development (1). When compared to their typically developing peers, children with CP usually show a shorter, and stiffer muscle belly that contributes to a decreased range of motion (ROM) (2). In the management of CP, treatment is often aimed at improving joint ROM and corresponding function. Invasive procedures, such as surgical lengthening of the muscle or tendon have been shown to increase musculotendinous lengths during gait (3). However, adverse effects of muscle lengthening surgery have also been reported. Medial hamstring lengthening in children with CP led to a shorter and smaller muscle belly (4), there is a risk of overcorrection (5), and the timing of surgery is essential for its success (6). Therefore, conservative approaches are preferred as a first treatment option. Stretching, as part of a physical therapy intervention, aims to increase muscle belly length and consequently improve function, delay the development of muscle contractures, and the need for surgery (7). It is assumed that stretching leads to an increase in muscle fascicle length by promoting an increase in sarcomeres in series (8), allowing the muscle fascicles to produce a force over a larger range of motion. However, in children with CP it is not known whether this process occurs after passive stretching. Fascicle length was shown not to change after a stretching intervention, where any changes in muscle morphology were attributed to the connective tissue (9).While a combined passive-stretching and active-movement training increased fascicle length (10), stretching with an ankle-foot brace actually decreased fascicle length (11). Furthermore, the influence of stretching on joint ROM is unclear (12), with some studies showing post-stretching improvement in ROM (9, 13) where others show no effects (14). Thus, a significant gap exists between the clinical rationale for stretching and the supporting evidence. Given that stretching exercises can be painful, demanding, and time consuming for children, their families and physiotherapists (15), stronger evidence is needed to improve their effectiveness and justify their application.

To find ways to increase effectiveness of stretching interventions, it is vital to understand how the muscle itself responds to stretch by joint rotation. It has been shown that in children with CP, medial gastrocnemius (GM) muscles are shorter (2, 16), Achilles tendon slack length is longer (17) and the relative stiffness of muscular to tendinous tissue is greater (18). These alterations result in a reduced stretching stimulus during a single stretch, as a greater amount of stretch is received by the more compliant in-series Achilles tendon. As such, it was shown that after a single bout of stretching in children with CP, ankle joint ROM increased acutely but no changes to the GM muscle properties were observed (19). A similar mechanism may limit the positive effect on muscle fascicle length after long term stretching interventions.

To resolve this issue, alternate solutions are required. Possibly, the amount of strain in the muscle fascicles could be increased by decreasing the relative stiffness of muscle to tendon. This could be achieved in different ways. For example, intramuscular botulinum toxin-A injections that temporarily reduces the symptoms of tonic discharge, are considered to provide a “window of opportunity” during which adjunctive interventions, such as casting and physiotherapy, are considered essential to improve, or at least prevent further worsening of, muscle structure (20). However, the effect of botulinum toxin-A is temporary and repeated injections can cause muscle atrophy (21), and damage (22, 23). Another way to alter the relative lengthening of muscle and tendon during passive stretch is to increase the stiffness of the tendon. It has been shown that tendon stiffness is adaptable and can increase with resistance training in adults (24), elderly (25), typically developing pre-pubertal children (26), and even in children with CP after dynamic movement training (10). Furthermore, resistance training has been shown to improve strength and muscle volume in children with CP (27, 28), indicating that spastic muscles can adapt in response to training. Therefore, it is reasonable to hypothesize that the tendons of spastic muscles are also adaptable to mechanical loading and they will increase their stiffness with resistance training. The effect of resistance training on muscle stiffness and length is minimal (29). In combination, this would reduce the relative stiffness of the muscle compared to the tendon and thus increase the stretching stimulus to the muscle when combined with stretching exercises.

To test this mechanistic theory, a combined strengthening and stretching intervention was designed. To increase Achilles tendon stiffness before starting the stretching exercises, the intervention group performed resistance training (heel-raises), while a control group performed upper limb strengthening, before both groups undertook an identical stretching intervention. We hypothesized that the combined intervention group, in contrast to the control group, would show an increase in fascicle length, which is indicative of muscle remodeling following treatment.

## Materials and Methods

### Participants

Twenty-two children with CP aged between 7 and 14 years old were recruited for participation in this study (median [IQR] age 9.6 [8.6, 10.5] years, Table 1). Children were excluded if they had received botulinum toxin-A injections to the lower limb muscles 6 months prior to testing, a baclofen pump, or any lower limb neuro- or orthopedic surgery. Inclusion criteria were: having been diagnosed with spastic cerebral palsy, a GMFCS level of I-III, the ability to perform at least one bi-lateral heel raise and aged between 7 and 14 years old. All children scheduled for an appointment at the gait laboratory of ^**^Blinded for review^**^ were assessed for eligibility. This study was carried out in accordance with the recommendations of the institutional as well as National Health Insurance (NHS) ethics committees (16/NW/0301). Written parental consent was obtained in accordance with *the declaration of Helsinki* and written assent was given by children. The study was registered on clinicaltrials.gov (NCT02766491).

**Table 1:** Characteristics of recruited participants.

	**Intervention (*n =* 12)**	**Control (*n =* 10)**
Age (years)	9.8 [9.3, 11.7]	9.0 [8.3, 10.1]
Sex	6 male, 6 female	7 male, 3 female
Diagnosis	6 Diplegia, 6 Hemiplegia	5 Diplegia, 5 Hemiplegia
GMFCS	7 × I, 5 × II	5 × I, 4 × II, 1 × III
Botulinum toxin-A injections >6 months before the study date [mean(range)]	0.33 (0–3)	0.5 (0–2)

*For age, median [IQR] values are shown*.

### Experimental Design

**Figure 1:**
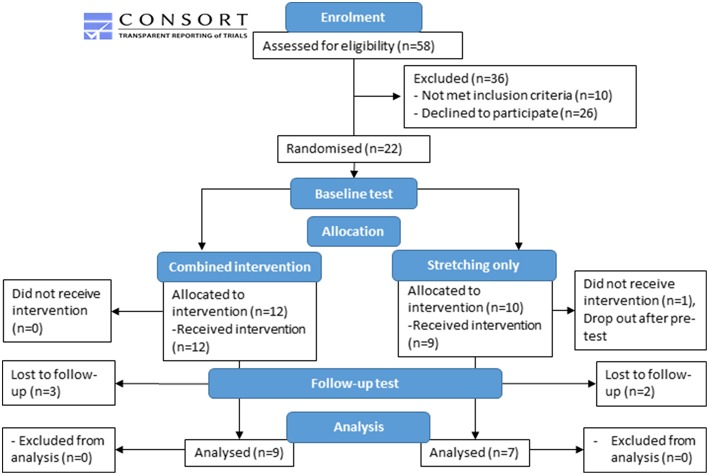
Flow diagram based on consort guidelines to show the experimental design.

All children scheduled for gait analysis were screened to fit the inclusion criteria, hence recruitment was an ongoing process. Therefore, children were randomly assigned to either the combined strengthening and stretching intervention (*n* = 12) or a control (stretching-only) group (*n* = 10) according to a computerized minimization algorithm (30). Allocation was concealed from participants until after baseline testing. At this point a computerized algorithm allocated the participants to a group. The computerized minimization algorithm was balanced for gender, age and GMFCS score which lead to an unequal number of participants allocated to the two groups (Figure 1). Participant characteristics at baseline were not different between groups and can be found in Table 1.

Children in the intervention group performed strengthening exercises of the calf muscle for 4 weeks, followed by 6 weeks of combined stretching and strengthening of the calf muscle. Children in the control group performed 6 weeks of stretching exercises of the calf muscles similar to the intervention group. To ensure the same amount of contact hours with the research team, the control group performed 4 weeks of strengthening exercises of the upper limb prior to the stretching. Measurements of tendon stiffness and muscle morphology were taken at three time points: before the start of the intervention (baseline), after 4 weeks of training (4-weeks) to assess the effect of the resistance exercises, and at completion of the intervention (10-weeks).

#### Training

Exercises were performed 4 times a week, of which the principal investigator supervised one session, and 3 were performed at home. All participants kept an exercise diary of their progress. In both groups, training focussed on the most affected side in terms of dorsiflexion ROM.

##### Strength-training

Standing unilateral heel raises were used to strengthen the ankle plantarflexor muscles. Frequency, intensity and repetitions were informed by the American College of Sport Medicine guidelines for progressive resistance training (31). The target was to complete 3 sets of 12 repetitions, in which volitional fatigue was reached at the end of each set. However, when children were unable to complete more than 6 repetitions with their own body weight, a fourth set was included to maintain total exercise volume. Participants who could not perform >6 unilateral heel raises at the start of the program started with bilateral heel raises and progressed to unilateral when possible. When participants could achieve more than 12 repetitions, exercises were progressed by adding weight to a rucksack worn on the participants back. The control group performed a similar protocol with seated biceps-curls with dumbbells as an exercise, targeting the elbow flexors. This group progressed by increasing the weight of the dumbbells.

##### Stretching

Stretching exercises of the calf muscle were performed after the resistance exercises and a couple of minutes rest in between. Stretching was performed either voluntarily by the participant (self-stretch) or having an external force applied by the parents. The method of stretching depended on capability (age and physical functioning) of the individual participant to perform the self-stretch. All stretches were held for 1 minute and repeated 10 times, with 30 s rest in between stretches. For the self-stretch, to gain the initial stretch position children were instructed to stand facing a wall, with the hands placed against the wall at shoulder height and the leg to be stretched placed behind the body. To perform the stretch, participants were asked to lean forwards and pull their hips toward the wall while pressing the heel of the stretched leg into the floor. For the externally applied stretch, children lay on their back on a mat on the floor with the parent positioned at the side of the participant. To gain the initial stretch position the leg was lifted with the knee flexed to 90°. To initiate a stretch, the hand was cupped around the heel with the palms of the hand flat against the foot. The ankle was slowly dorsiflexed by applying pressure against the plantar surface of the foot. When in a maximal dorsiflexed position, the knee was slowly guided into maximal extension. To assure a sufficient stretch stimulus in both groups, the participants and parent/career were properly instructed to perform an overpressure at the end of the ROM to feel the rise in resistance. Children were assured to feel a slightly uncomfortable stretching sensation in the muscle.

### Measurement Protocol

**Figure 2:**
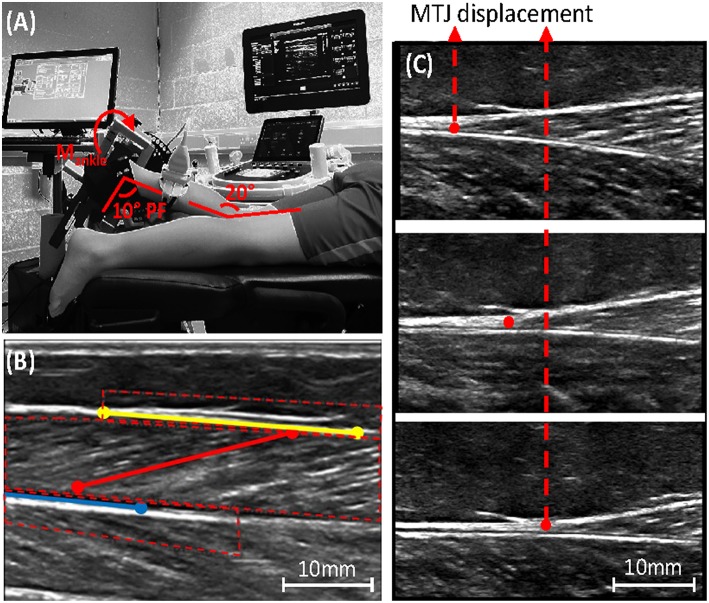
**(A)** Experimental set-up, showing leg placement in the dynamometer combined with ultrasound placement over the medial gastrocnemius muscle-tendon-junction (MTJ). **(B)** Ultrasound image of the medial gastrocnemius with the defined range of interest needed to track fascicle length (red), the upper (yellow), and lower (blue) aponeurosis; see online version for color. **(C)** Ultrasound image showing medial gastrocnemius MTJ displacement during muscle contraction.

Tibia length was measured as the distance from the tibiofemoral joint space to the lateral malleolus. Participants lay prone on a bed with the hip fully extended and the lower leg supported on an inclined cushion such that the knee was ~20° flexed. The foot was secured to the footplate of a dynamometer (Humac Norm CSMI, MA, USA) with the help of a custom-made arch support that ensured heel contact with the footplate during ankle rotation. The axis of rotation of the dynamometer was aligned with the lateral malleolus during passive movement (Figure 2A). Range of motion in both directions was determined by manually rotating the ankle to the point where either the participant indicated the threshold or the examiner felt the joint reached the end of passive movement. Subsequently the stops of the dynamometer were set to this ROM. Surface EMG (BioNomadix, Biopac systems, UK), placed on the middle of the muscle belly as defined with ultrasound, was used to collect signals at 1,600 Hz from the lateral gastrocnemius, to assure that no spastic response was evoked during the testing protocol. Ankle torque and joint angle were measured at 200 Hz by a dynamometer and collected in Acknowledge software (Biopac systems, UK) synchronously with EMG. The net joint plantarflexion torque was corrected for the torque caused by gravity on the weight of the footplate. The weight of the foot was considered negligible compared to the weight of the footplate.

#### Passive Muscle Structure

A B-mode ultrasound scanner (Phillips EPIQ7, The Netherlands) was used to measure muscle structure. The ultrasound probe was placed over the mid-belly of the medial gastrocnemius muscle. Guidance regarding probe alignment was adhered to for minimizing measurement errors (32). As the primary outcome measures, fascicle and muscle length was measured at rest, with the foot hanging off the edge of the bed and the knee positioned at 20° flexion. To exclude any effect of viscoelastic and thixotropic changes in the muscle, it was assured that the muscle was at rest by visual inspection of the EMG signal for at least 30 s before the measurement was taken. Fascicle lengths were also measured during a continuous passive movement applied by the dynamometer, from maximal plantarflexion to maximal dorsiflexion performed at 7.5°/s. Four separate images of resting fascicle length were taken and the passive movement was repeated four times. There was a minimum of 10 s of rest between individual repetitions.

All ultrasound images were anonymized before analysis to blind the assessor to the allocation. During the passive movements, a semi-automated tracking software (33, 34) was modified such that the fascicle as well as the superficial and the deep aponeurosis could be tracked. Fascicle length was calculated by extrapolating the fascicle to the intersection point with the aponeuroses (Figure 2B). For each trial, the ankle angle at which the fascicles started to lengthen was determined visually from the plotted fascicle vs. ankle angle relationship and from the ultrasound video. All measurements were averaged over the four trials.

Muscle belly and Achilles tendon lengths were measured at 4 static positions: maximal dorsiflexion (as defined above); maximal plantarflexion; a common ankle angle (10° plantarflexion) and resting ankle angle (as defined above). At each joint angle, the most superficial point on the posterior of the medial femoral condyle, the most distal point of the medial gastrocnemius myotendinous junction (MTJ), and the most distal point of the attachment of the Achilles tendon on the calcaneus were identified with ultrasound and marked on the skin with help of a thin metal rod placed between the skin and the ultrasound probe (35). Muscle and tendon lengths were measured with a segmometer as the linear distances between the medial femoral condyle and the MTJ; and between the MTJ and the attachment on the calcaneus, respectively.

#### Achilles Tendon Stiffness

Tendon stiffness was calculated as the gradient of the force-elongation curve during a maximal voluntary isometric plantarflexion contraction (MVC). Two repetitions were performed of the MVC with at least 1 min rest in between. Tendon elongation was measured with ultrasound as the displacement of the medial gastrocnemius MTJ during the MVC trials (Figure 2C). The ultrasound probe was secured perpendicular to the skin above the MTJ, oriented along the line of action of the muscle. An echo-absorptive strip was placed over the skin above the muscle to confirm that the ultrasound probe did not move relative to the skin during the measurement. Displacement of the MTJ was manually tracked in every image frame, twice for each contraction, using ImageJ imaging software (NIH, USA). Achilles tendon force was calculated by: *F*_*tendon*_ = *M*_*joint*_/*MA*_*AT*_, where M_joint_ is the joint moment as measured by the dynamometer and MA_AT_ is the Achilles tendon moment arm estimated for the specific joint angle based on the predictive equation derived from Kalkman et al. (36).

Tendon stiffness was calculated by fitting a second-order polynomial (R^2^: 0.96 ± 0.09) through each full force-elongation curve and differentiating at a force level that could be achieved both pre- and post-intervention (37). This method was chosen to account for confounding factors such as the absolute maximal force and the ability to activate the muscle. The calculated tendon stiffness was averaged over the different repetitions obtained from the two trials.

Despite practice, some children found it difficult to perform these slow contractions smoothly. These children were asked to produce their MVCs at their preferred speed. Because the rate of force development was markedly lower than in adults (38), the resolution of our force elongation curves created with an ultrasound sampling frequency of 15–40 Hz, depending on the ultrasound settings, resulted in a minimum of 30 data points per contraction, which was considered adequate for reliable data capture.

### Statistics

Parameters were checked to be normally distributed using the Shapiro-Wilk test and by inspection of the q-q plots. None of the variables of interest followed a normal distribution. Between group comparisons of the change in outcome measure from baseline to 10 weeks were made for MVC, tendon stiffness, muscle and tendon length, and resting fascicle lengths using a Mann-Whitney *U*-test. Baseline measures were compared between groups using a Mann-Whitney *U*-test. A *p*-value of ≤ 0.05 was considered significant. For all parameters median and 95% confidence intervals (CI) were calculated using bootstrapping. All statistical analyses were performed in Matlab 2017a.

## Results

A total of 16 children completed the intervention and all the testing sessions. Because this study is an efficacy trial, only children who completed the intervention were included in the analysis. For those children completing the study, there were differences between the intervention and the control group in age, height and mass at baseline (Table 2) due to unequal drop-out rates. However, there were no significant differences in absolute or normalized resting fascicle length (Median [IQR]: intervention: 35.8 [30.1, 45.7] mm, control: 27.9 [27.0, 38.1] mm, *p* = 0.11), plantarflexion MVC (intervention: 5.8 [3.1, 18.8] Nm, control: 8.1 [3.7, 17.2] Nm, *p* = 0.33) or tendon stiffness (intervention: 30.1 [11, 64] N/mm, control: 27.8 [15.3, 31.4] N/mm, *p* = 0.32) between groups at baseline.

**Table 2:** Characteristics of participants completing the intervention.

	**Intervention (*n =* 9)**	**Control (*n =* 7)**
Age (years)	11.1 [9.4, 12.7]	8.5 [7.8, 10.1]*
Height (cm)	145 [131.5, 157]	125.5 [122, 136]*
Mass (kg)	35.5 [32, 43.5]	24 [21.5, 29]*
Sex	4 male, 5 female	5 male, 2 female
Diagnosis	5 Diplegia, 4 Hemiplegia	3 Diplegia, 4 Hemiplegia
GMFCS	5 × I, 4 × II	3 × I, 3 × II, 1 × III

Median [IQR] values are shown;

* *Significant difference at p < 0.05*.

Of the nine participants in the intervention group, two began training with bilateral heel raises, both progressed to uni-lateral heel raises in the second week. Significant increases in the number of heel raises achieved as well as the load added to the backpack (max 10% of body mass) were found between baseline and week 10 for the whole group (Table 3). Adherence to the training sessions was close to perfect. Maximal plantarflexion MVC as assessed with the dynamometer increased more in the intervention group after 10 weeks (250% vs. −20%, *p* = 0.009, Table 3).

**Table 3:** Training related measures.

	**Baseline**	**Week-10**
Uni-lateral repetitions (#)	8 (0–10)	12 (12-12)*
Added load (kg)	0 (0–0)	2.6 (0–6)*
MVC (Nm)	5.8 [3.1, 18.8]	14.5 [10.8, 29.1]*

* *Significant difference between week-10 and baseline measurements (p < 0.001). For repetitions and load, median (range) values are shown. For maximal voluntary contraction (MVC), median [IQR] values are shown*.

Tendon stiffness increased more in the intervention group compared to the control group after 10 weeks (intervention: 13.6 N/m, control: 1.5 N/m, *p* = 0.04, Table 4). After 4 weeks there was no significant difference in the change in tendon stiffness between groups (*p* = 0.28).

**Table 4:** Muscle tendon parameters.

	**Control**				**Intervention**			
	**Baseline median [IQR]**	**4-weeks median [IQR]**	**10-weeks median [IQR]**	**Change baseline to 10-weeks median [95% CI]**	**Baseline median [IQR]**	**4-weeks median [IQR]**	**10-weeks median [IQR]**	**Change baseline to 10-weeks median [95% CI]**
**At rest**								
Fascicle length (mm)	27.9 [27.0, 38.1]	30.9 [24.4, 41.6]	29.1 [25.3, 37.6]	−0.5 [−2.7, 1.6]	35.8 [30.1, 45.7]	34.7 [31.7, 40.3]	39.8 [31.6, 50.0]	2.2 [1.3, 4.3]*
Ankle angle (°)	−32.5 [−18, −44]	/	−36 [20, −47.5]	0 [−10, 3]	−30 [−35, −26]	/	−26.5 [−33, −25]	3.5 [−4.5, 5]
Muscle length (mm)	14.2 [12.7, 15.2]	/	14.0 [12.6, 14.9]	−0.1 [−0.35, 0.3]	17.6 [15.7, 18.9]	/	17.9 [15.9, 19.8]	0.15 [−0.1, 0.5]
Tendon length (mm)	15.8 [13.8, 16.3]	/	15.5 [14.6, 16.3]	0.05 [−0.45, 0.5]	16.5 [15.2, 20.4]	/	18.4 [15.8, 20.3]	0.15 [−0.35, 0.55]
**At maximal DF**								
Fascicle length (mm)	41.2 [33.1, 52.7]	/	40.7 [35.2, 55.4]	2.7 [−0.49, 3.22]	50.9 [35.0, 65.1]	/	52.5 [44.0, 69.7]	6.5 [−0.77, 10.2]
Ankle angle (°)	17 [−26, 30]	/	22 [−9, 27]	5 [2, 10]	7.5 [−4, 17]	/	17.5 [9, 30]	7.5 [3, 15]
Muscle length (mm)	15.1 [13.5, 16.1]		15.5 [13.9, 16.8]	0.65 [0.4, 0.7]	18.9 [17.0, 20.2]	/	19.3 [17.7, 20.5]	0.45 [0.05, 0.68]
Tendon length (mm)	16.1 [13.9, 16.6]		15.5 [14.6, 15.9]	−0.15 [−1.0, 0.2]	17.3 [13.8, 21.1]	/	17.3 [14.8, 20.1]	−0.18 [−0.85, 1.05]
**Tendon stiffness**								
Common force (N/mm)	27.8 [15.3, 31.4]	21.0 [13.0, 28.9]	30.2 [13.2, 43.1]	0.8 [−5.7, 13]	30.1 [11, 64]	45.9 [29.2, 63.1]	45.9 [25.2, 74.7]	13.6 [9.9, 17.7]*

*Values are median and IQR for absolute values or median and 95% confidence intervals (CI) for change between baseline to 10-weeks; Change between baseline and 10-weeks is shown as the median of the paired difference*.

* *Significant difference between intervention and control in the change from baseline to 10-weeks*.

**Figure 3:**
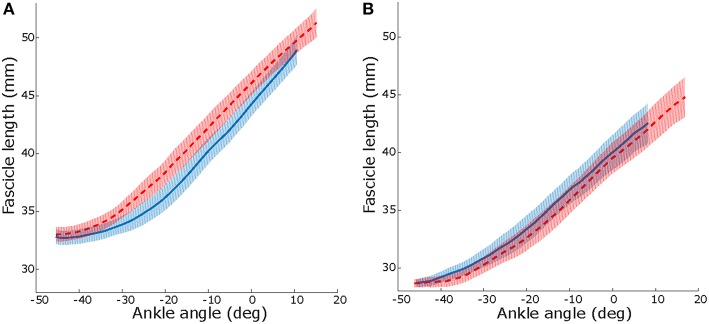
Median and IQR over all participant, lengthening profiles of muscle fascicles vs. ankle angle during a passive joint rotation in the intervention **(A)** and control **(B)** group. Negative angles reflect plantarflexion position. Solid blue: baseline, dashed red: after 10 weeks of intervention. See online version for color.

After 10 weeks, resting fascicle length increased more in the intervention group than in the control group (2.2 vs. −0.5 mm; *p* = 0.02; Table 4). Changes in resting muscle length were not different between groups (*p* = 0.24). The ankle angle at which resting length was assessed did not change (*p* = 0.670; Table 4). Maximal dorsiflexion angle increased similarly in both groups between baseline and 10-weeks (*p* = 0.78; Table 4). During passive joint rotation, the angle at which muscle fascicles started to lengthen shifted toward a more plantarflexed position in the intervention group (baseline to 10-weeks intervention: −6 [−12, 0]°, control: 5 [0, 12]° *p* = 0.03, Figure 3). Muscle and tendon lengths did not change after the intervention at any ankle angle (max. dorsiflexion, 10° plantarflexion, resting, max. plantarflexion) and there were no differences between control and intervention group (Table 4). Averaged lengthening profiles of all muscle fascicles over the ROM (with negative angles reflecting plantarflexion) are visualized in Figure 3.

## Discussion

The present study shows that in response to a combined progressive resistance training and stretching programme, tendon stiffness increased in children with CP compared to a control group. As a result of this increase in tendon stiffness, the stretching stimulus to the muscle would be increased. Consequently, in this combined intervention group, resting fascicle length also increased more than in a stretching-only group. This provides proof of principle that a combined resistance training and stretching intervention may be useful in increasing muscle fascicle length of children with CP. Apart from an increased joint ROM, this could lead to a greater length range over which the muscle can produce force, thereby enhancing physical function. Furthermore, since muscle contractures develop over time as children grow (39), an increase in fascicle length may prevent the development of contractures and therefore delay or remove the need for orthopedic surgery. The children that participated in this study were generally higher functioning (GMFCS I-III), therefore this intervention to increase strength, tendon stiffness and ROM might be particularly useful for children who are able to undertake volitional plantarflexion contractions.

Increases in ROM have been shown in response to stretching in children with CP (9), while others report no changes in flexibility (40–42). After 16 weeks of stretching by means of ankle-foot braces, it was shown that while ROM improved, fascicle length actually decreased (11). Similarly, in our study, 6 weeks of performing only stretching exercises (control group) seems to increase ankle ROM by 5° as indicted by a 95% CI not crossing zero, but this was not accompanied by a change in fascicle architecture. Due to the experimental setup, fascicle lengths were only measured at the mid-belly of the muscle, therefore it is assumed that this extends to the distal and proximal parts of the muscle. In our study design, as in the clinical practice of stretching, the examiner manually stretched the ankle to its end ROM, defined as the “end-feel” (43). The position at which this end-feel occurs may depend on different factors including: pain tolerance, warm-up of the muscle or acquaintance between the patient and the examiner. Given the lack of muscle remodeling, we can hypothesize that the children experienced an increased stretch tolerance after the intervention. Although we cannot exclude potential alterations in the properties of the extra-cellular matrix, which might play a role in the development of contractures (44). However, changes in stretch tolerance is consistent with the mechanism previously identified to explain the acutely increased ROM after a single bout of stretching (19). Therefore, it is unlikely that stretching exercises alone improve function or delay the development of contractures.

In this study, stretching exercises were preceded by 4 weeks of resistance training to increase Achilles tendon stiffness. Large increases in tendon stiffness (47%) were observed after these 10 weeks of resistance training. Similar increases of 65% and 51% in tendon stiffness have been observed in older and younger adults, respectively. In both cases, increases were attributed to a change in the material properties of the tendon and not due to tendon hypertrophy (25, 45).In the current study, we cannot make this distinction since we did not measure the cross-sectional area of the tendon.

In combination with the stretching exercises, our treatment led to a median increase of 2.2 mm in resting fascicle length. This resting position was measured at a constant ankle joint torque of approximately 0 Nm (only the weight of the foot acting on it), it can be assumed that the muscle was near its slack length at this position. However, a direct measure of fascicle slack length *in vivo* is not possible. Interestingly, muscle belly length at a resting ankle angle did not change after the intervention. This might be caused by a change in pennation angle due to resistance training (46) or alternate changes at the distal or proximal part of the muscle belly. The increase in fascicle length, could be caused both by an increase in the number, or length of the sarcomeres acting in series. In healthy muscles, the passive resting length of a sarcomere is mainly determined by the large molecule titin (47). Therefore, an increase in sarcomere slack length should be caused by an alteration in mechanical properties or length of the titin molecule. There is no definitive data to show that these are altered in spastic muscles, indicated by a similar molecular mass in CP and control biopsies (44) or as a result of training. However, titin isoforms have been found to be different between individual muscles in rabbits (48) and altered by disease in heart muscle (49). Investigations into the alterations that occur in titin in spastic skeletal muscle, and after training, are warranted. Alternatively, the increased fascicle length can be caused by an addition of sarcomeres in series. It has previously been hypothesized that muscles of children with CP are unable to add sarcomeres in series in response to chronic stretch (bone growth) (50) as has been shown to be the case in animal models (8). Studies showing a reduction in the number and function of satellite cells in spastic muscle (51, 52) may support this hypothesis. However, a recent study showed that, at least in a mouse model, when muscles underwent satellite cell depletion, sarcomeres could still be added in series in response to chronic stretch (53). With a clear conclusion still lacking, it is feasible that the increased fascicle length in this study was caused by an increase in the number of sarcomeres in series, especially since the fascicle length—ankle angle relationshipwas unaltered. Nonetheless, whichever of these scenarios causes the increased fascicle length seen after a combined strengthening/stretching intervention, the current data manifest that a remodeling of muscle structure in response to mechanical loading (stretching) is possible in children with CP, as long as the in-series tendon is stiff enough to effectively transmit the stretching stimulus to the muscle.

A number of limitations should be acknowledged. Initially we aimed to recruit 30 participants for the study. However, challenges of recruitment meant we only reached 22 participants starting the trial. Of these, 16 participants completed the trial, the reason for dropout was not related to the intervention in any way. There was a significant difference between groups who completed the intervention in age, height and body mass. This was caused by drop-out of the 3 youngest children from the intervention group. Even though resistance training is recommended for children aged over 7 years (31), it might be unsuitable for young children who lack matured motor control skills. However, since outcome measures did not differ between groups at baseline we do not expect this to have influenced our results. Due to the experimental setup, during the measurements, the examiner could not be blinded to the intervention the children received. However, all ultrasound images were anonymized for participant and time point before the analysis. We did not assess the effect of isolated strength training on muscle structure. We cannot exclude that due to the nature of the strength exercises (children put their foot flat on the floor) a stretch was induced to the muscle already during the strengthening exercises. However, previous results show no changes in muscle length due to a pure resistance training protocol in adolescents with CP (29). On the other hand, the increase in tendon stiffness cannot be attributed to the resistance training alone, since the change in stiffness was only detected to be significant after 10 weeks. However, previous studies indicate that stretching does not influence tendon stiffness (9). Also, some assumptions were made in the calculation of tendon stiffness. Co-contraction of the antagonist muscle was not accounted for when calculating tendon force because for most children it was too difficult to perform a required dorsiflexion contraction. This would cause an underestimation in tendon stiffness. It might be expected that the amount of co-contraction would decrease after strengthening (54). Therefore, our calculation of tendon stiffness may be more underestimated after the intervention than at baseline. As a consequence, accounting for co-contraction would only enlarge the increase in tendon stiffness we report. Also, because not all children were able to perform a slow plantarflexion contraction, we could not confirm the strain rate of the tendon to be constant during the MVC. It has been shown that strain rate influences the calculation of tendon stiffness (55). However, additional analysis indicated no significant differences in strain rate at 10-weeks vs. baseline (10-weeks: 4.43 ± 2.3 mm/s, baseline: 4.06 ± 1.6 mm/s, *p* = 0.17). Therefore, we do not expect this to have influenced our results. It was ensured that children were relaxed for 30s before taking the measurement, since more was not feasible to achieve. Therefore, outcomes of resting lengths are potentially confounded by muscle thixotropic properties. Also, unmeasured torque data during passive movements could potentially confound these measures. Finally, altered active properties of the soleus muscle could have contributed to the increased stiffness of the Achilles tendon since measurements were taken from the MTJ of the medial gastrocnemius. Whichever the cause, the increased Achilles tendon stiffness, would still have the same effect on the medial gastrocnemius muscle.

To conclude, this study documented that a combined resistance training and passive stretching intervention can increase muscle fascicle length in children with CP. This is an important finding because it not only demonstrates that remodeling of muscle structure is possible with non-invasive interventions in spastic CP, but also provides proof of principle that this combination of strengthening and stretching is an effective way to improve muscle architecture. Further long-term studies are required to determine the physiological pathways behind muscle remodeling in spastic muscles and to assess whether these changes in the muscle are maintained and could help to prevent contractures in children with CP at a later age.

## Data Availability

The dataset generated for this study can be found on figshare (https://figshare.shef.ac.uk/s/1f6a6d565083e49cab1e).

## Ethics Statement

This study was carried out in accordance with the recommendations of the institutional as well as NHS ethics committees. Written parental consent was obtained in accordance with the declaration of Helsinki and written assent was given by children. The study was registered on clinicaltrials.gov (NCT02766491).

## Author Contributions

All authors have read the manuscript and agreed to its being submitted for publication. All individuals listed as authors meet the appropriate authorship criteria, nobody who qualifies for authorship has been omitted from the list. In brief, all authors contributed to conception and design of the research. BK, GH to data acquisition. BK to data analysis. All authors to the interpretation of the results. BK drafted the manuscript. All authors edited and revised the manuscript.

### Conflict of Interest Statement

The authors declare that the research was conducted in the absence of any commercial or financial relationships that could be construed as a potential conflict of interest. The reviewer HM declared a shared affiliation, though no other collaboration, with one of the author LB-O.
